# Impact of high-risk and low-risk human papillomavirus infections on the male genital tract: effects on semen inflammation and sperm quality

**DOI:** 10.3389/fcimb.2024.1420307

**Published:** 2024-08-23

**Authors:** Carolina Olivera, Daniela A. Paira, Andrés Olmedo, José J. Olmedo, Andrea D. Tissera, Rosa I. Molina, Rubén D. Motrich, Cecilia G. Cuffini, Virginia E. Rivero

**Affiliations:** ^1^ Federation of Clinical Immunology Societies (FOCIS) Center of Excellence Centro de Inmunología Clínica de Córdoba (CICC), Córdoba, Argentina; ^2^ Centro de Investigaciones en Bioquímica Clínica e Inmunología (CIBICI)-CONICET, Facultad de Ciencias Químicas, Universidad Nacional de Córdoba, Córdoba, Argentina; ^3^ Dirección de Asistencia Social del Personal Universitario (DASPU), Universidad Nacional de Córdoba, Córdoba, Argentina; ^4^ Fundación Urológica Córdoba para la Docencia e Investigación Médica (FUCDIM), Córdoba, Argentina; ^5^ Laboratorio de Andrología y Reproducción (LAR), Córdoba, Argentina; ^6^ Instituto de Virología Dr. Jose Maria Vanella, Facultad de Ciencias Médicas, Universidad Nacional de Córdoba, Córdoba, Argentina

**Keywords:** HPV, high-risk and low-risk HPV genotypes, semen cytokines, male genital tract, ROS

## Abstract

Human Papillomavirus (HPV), a prevalent sexually transmitted infection, comprises high-risk (HR-HPV) and low-risk (LR-HPV) viruses, the former posing a high risk for developing malignancies whereas the latter mainly for benign warts. Despite increasing awareness of HPV’s impact on men’s health, the influence of HR-HPV and LR-HPV urogenital infections on male fertility potential remains uncertain. This study aimed to investigate whether male urogenital infection with HR- or LR-HPV associates with impaired sperm quality, oxidative stress, and inflammation. A total of 205 male patients attending an urology clinic were enrolled. Semen samples were analyzed for HPV using PCR and genotyped by RFLP. Semen quality was evaluated following WHO guidelines. Semen leukocytes, reactive oxygen species (ROS), and sperm viability were analyzed using flow cytometry. HPV was detected in 19% (39/205) of semen samples. HR-HPV infections were more prevalent, with HPV-16 being the most frequent genotype. Neither HR-HPV nor LR-HPV were associated with significant alterations in routine sperm quality parameters. However, HR-HPV+ individuals showed significantly higher levels of sperm necrosis and exhibited increased proportions of ROS+ spermatozoa compared to LR-HPV+ or control individuals. Furthermore, no significant semen inflammation was detected in patients infected with either HR-HPV or LR-HPV, and unexpectedly reduced semen leukocytes and inflammatory cytokines (IL-6 and IL-1β) were observed in HR-HPV+ patients compared to controls. These observations underscore the importance of comprehensive HPV screening, including genotyping, in urology and fertility clinics to understand the progression of the infection, potential adverse effects on reproductive health, and the oncogenic risks involved.

## 1 Introduction

Human Papillomavirus (HPV) is a highly prevalent sexually transmitted infection that has long been associated with the development of cervical cancer (CC) in women primarily (Darvishi et al., 2023; Plotzker et al., 2023). HPV comprises a diverse family of viruses, with certain types classified as high-risk (HR-HPV) due to their association with an elevated risk of developing malignancies, including cervical, anal, and oropharyngeal cancers, whereas others classified as low-risk (LR-HPV), typically causing benign genital warts (Darvishi et al., 2023; Plotzker et al., 2023). Identifying whether a woman carries LR-HPV or HR-HPV genotypes is crucial for several reasons. As well known, HR-HPV genotypes can cause CC and may warrant more frequent follow-up and testing to detect cell abnormalities or cancer at an early stage (Plotzker et al., 2023). In addition, clinical management of patients vary according to the virus genotype detected, being monitoring and treatment more aggressive for some genotypes (Andújar-Sánchez, 2023; Plotzker et al., 2023). Moreover, HPV genotype information allows for personalized risk assessment both for patient and their sexual partner(s) care, which can help preventing further spread of the virus and to take informed decisions about reproductive health (Plotzker et al., 2023).

Although, studies of HPV‐related disorders have previously focused almost exclusively on females, interest in HPV urogenital infection in males is now expanding and recent research has shed light on its prevalence and potential consequences in males as well (Bruni et al., 2023; Garolla et al., 2024). HPV infection in men is common, but its association with cancer has been less extensively studied than in women (Sichero et al., 2019). HPV infection in men is considered to be transient, with a main clinical expression being warts in external genitalia (Capra et al., 2015). HPV presence has been documented in different specimens from the male genital tract. Indeed, HPV DNA has been detected in external genitalia, semen, urethra, prostate, vas deferens, epididymis, and testis. However, it is still controversial whether the detection of HPV in the upper male genital specimens is due to contamination from genital skin and mucosa (Giuliano et al., 2007; Laprise et al., 2014; Capra et al., 2015; Fedder et al., 2019). Both HR and LR-HPV genotypes have been identified in semen and other male urogenital specimens, with around one in five men infected with at least one type of HR-HPV (Bruni et al., 2023). In fact, it has been shown that HPV virions can bind to different sites at the equatorial region of the sperm head surface (Pérez-Andino et al., 2009). Accumulating evidence suggests that HPV can exert a range of effects on the male reproductive system; however, research focused on elucidating the consequences of seminal HPV infection has provided conflicting results (Lyu et al., 2017; Moreno-Sepulveda and Rajmil, 2021). While some studies associated this infection with decreased motility, altered morphology, sperm DNA fragmentation (Garolla et al., 2011; Moghimi et al., 2019; Capra et al., 2022), and adverse reproductive outcomes (Yang et al., 2013; Tramontano et al., 2023), other studies did not confirm these findings (Schillaci et al., 2013; Golob et al., 2014). Another aspect that adds to this complexity is the potential presence of other uropathogens coinfections that could also affect sperm quality (Fasciana et al., 2021). In addition to the contradictory findings regarding the impact of HPV infection on semen quality, scarce to absent data elucidating whether HPV infection in males is associated with inflammatory markers such as elevated cytokine levels, reactive oxygen species (ROS), and/or an increase in leukocyte populations in semen has been reported (Pérez-Soto et al., 2021; Das et al., 2023).

Despite growing recognition of the implications of HPV urogenital infection on men’s health, the specific impact of HR and LR-HPV infections remains a topic of significant interest and largely understudied. The aim of the present work is to analyze sperm quality as well as semen inflammation (including seminal levels of cytokines, ROS, and leukocyte subsets) in men with urogenital infection with low-risk (LR) or high-risk (HR) HPV genotypes.

## 2 Materials and methods

### 2.1 Study design, subjects, and samples

This cross-sectional study enrolled 205 adult males attending an urology and andrology clinic from 2018-2021. Eligible participants were males aged ≥18 years old, who underwent a semen analysis as part of their initial fertility assessment, or due to lower urinary tract symptoms or as a routine control without presenting any complaints related to infertility. Exclusion criteria: HPV vaccination, vasectomy, azoospermia, significant varicocele, or documented exposure to environmental pollutant such as pesticides; drug, alcohol, or marijuana consumption; fever or antibiotic treatment. The study was carried out in accordance with the Code of Ethics of the World Medical Association (Declaration of Helsinki) standards and the Argentinian legislation for protection of personal data (Law 25326). The experimental protocol was approved by the Institutional Ethics Committee from Centro Médico Oulton-Romagosa, Córdoba, Argentina (RePIS #3625). All subjects signed a written informed consent form agreeing to participate in the study. Subjects were subdivided in three groups as follow: control group: individuals negative for all analyzed uropathogens without leukocytospermia as described in the following section; HR-HPV patients: patients positive for urogenital infection with intermediate or high oncogenic risk HPV genotypes; LR-HPV patients: patients positive for urogenital infection with low oncogenic risk HPV genotypes.

### 2.2 Semen collection

To reduce contamination and improve sample quality for analysis, patients were instructed to thoroughly cleanse their hands and penis prior to semen collection. Samples were collected through masturbation after 3-5 days of sexual abstinence, ejaculated directly into a sterile container, and delivered to the laboratory within 1 hour. The samples were liquefied at 37°C for 20 minutes before semen quality analysis. Recovered seminal plasma was stored at -80°C until further use.

### 2.3 Uropathogens screening and HPV genotyping

The detection of Human Papillomavirus (HPV), *Ureaplasma urealyticum*, *Mycoplasma hominis*, *Chlamydia trachomatis*, herpes simplex virus (HSV) 1, HSV2, *Mycoplasma genitalium*, *Treponema pallidum*, *Neisseria gonorrhoeae*, and *Trichomonas vaginalis* were analyzed by polymerase chain reaction (PCR) in DNA purified from semen specimens using specific primers, as previously described (Paira et al., 2023). The presence of *Escherichia coli*, *Enterococcus faecalis*, *Proteus mirabilis*, *Enterobacteriaceae*, *Pseudomonas* spp., *Streptococcus* spp., *Staphylococcus* spp., *Corynebacteriaceae*, and *Candida* spp. was analyzed by semen culture as previously described (Paira et al., 2023). In addition, semen samples with HPV-specific DNA amplification using the consensus primers MY11/MY09 which target the L1 region of the viral genome, were further analyzed by PCR-Restriction Fragment Length Polymorphism (PCR-RFLP) as previously described (Bernard et al., 1994; De Villiers et al., 2004; Olivera et al., 2021). In brief, PCR amplicons were subjected to digestion with 7 restriction enzymes (Bam HI, Dde I, Hae III, Hinf I, Pst I, Rsa I y Sau IIIA) and subsequently subjected to electrophoretic analysis. After that, the size of each fragment was determined and genotypes assigned after comparing the patterns of molecular weights of fragments for each HPV genotype as previously described (Bernard et al., 1994; De Villiers et al., 2004).

### 2.4 Semen analysis

Semen analysis was performed at least twice in each individual sample according to the World Health Organization Semen Analysis Manual 5^th^ edition (2010) (World Health Organization, 2010). Routine sperm parameters were assessed in at least 200 spermatozoa per sample by 2 operators, rendering a total of 400 scored spermatozoa.

### 2.5 Sperm apoptosis/necrosis assessment by flow cytometry

Sperm apoptosis/necrosis was assessed immediately after semen liquefaction by annexin V (AnV)/propidium iodide (PI) staining and flow cytometry as previously described (Puerta Suarez et al., 2017).

### 2.6 Semen leukocyte analysis by flow cytometry

The assessment of leukocytes subsets in semen was performed as previously described (Paira et al., 2023). Single-cell suspensions were obtained after centrifuging 100 µl of semen specimen for 5 min at 2000 rpm to remove seminal plasma and resuspending the cells in ice cold FACS Buffer (10% FBS supplemented PBS). Cell suspensions were stained with fluorescent labeled antibodies specific to human CD45, CD3, CD4, CD8, and CD19 (BioLegend, San Diego, CA, USA) and to CD14, CD11c and CD11b (eBioscience) and analyzed on FACS Canto II cytometer. The entire sample was acquired in order to make a ratio of CD45+ cells per mL of semen. Data were analyzed using the FlowJo 7.6 software (Tree Star, Ashland, OR, USA).

### 2.7 Cytokine quantification

IL-8, TNF, IL-1β, IL-6, IFNγ, IL-10, and IL-17A concentrations in seminal plasma were analyzed using enzyme-linked immunosorbent assay (ELISA)–specific kits according to the manufacturer’s instructions. IL8-, TNF, IL-6, IFNγ, and IL-10 were respectively quantitated by the BD OptEIA specific ELISA sets (BD Biosciences Pharmingen, San Diego, CA, USA). IL-17A and IL-1β were respectively assayed by ELISA Ready-SET-Go specific kits (eBioscience, San Diego, CA, USA). Samples were analyzed at least in triplicates and results expressed as pg/mL. In the case of IL-8 quantitation, seminal plasma samples were assayed diluted 1/20.

### 2.8 Assessment of sperm reactive oxygen species production

Sperm ROS production was analyzed as previously described (Puerta Suarez et al., 2017). In brief, intracellular ROS levels were evaluated by flow cytometry using a cell-permeable probe 2’,7’-dichlorodihydrofluorescein diacetate (Dcfh-DA, 1μM; Sigma-Aldrich). This method enables a sensitive quantification of ROS in response to oxidative metabolism. Propidium iodide (PI, Molecular Probes Inc., The Netherlands) was used in conjunction with Dcfh-DA as a supravital stain (final concentration: 12μM).

### 2.9 Statistical analysis

Statistical analysis was performed using the GraphPad Prism software, version 9.0 (GraphPad Inc., La Jolla, CA, USA) and the SPSS Statistics for Windows software, version 25.0 (IBM, Armonk, NY, USA). Data distribution was assessed by the Shapiro– Wilk test. Data were analyzed using the Mann–Whitney tests. Differences were considered statistically significant when *p* < 0.05.

## 3 Results

### 3.1 Prevalence and characterization of HPV urogenital infection in men attending a urology and andrology clinic

**Figure 1:**
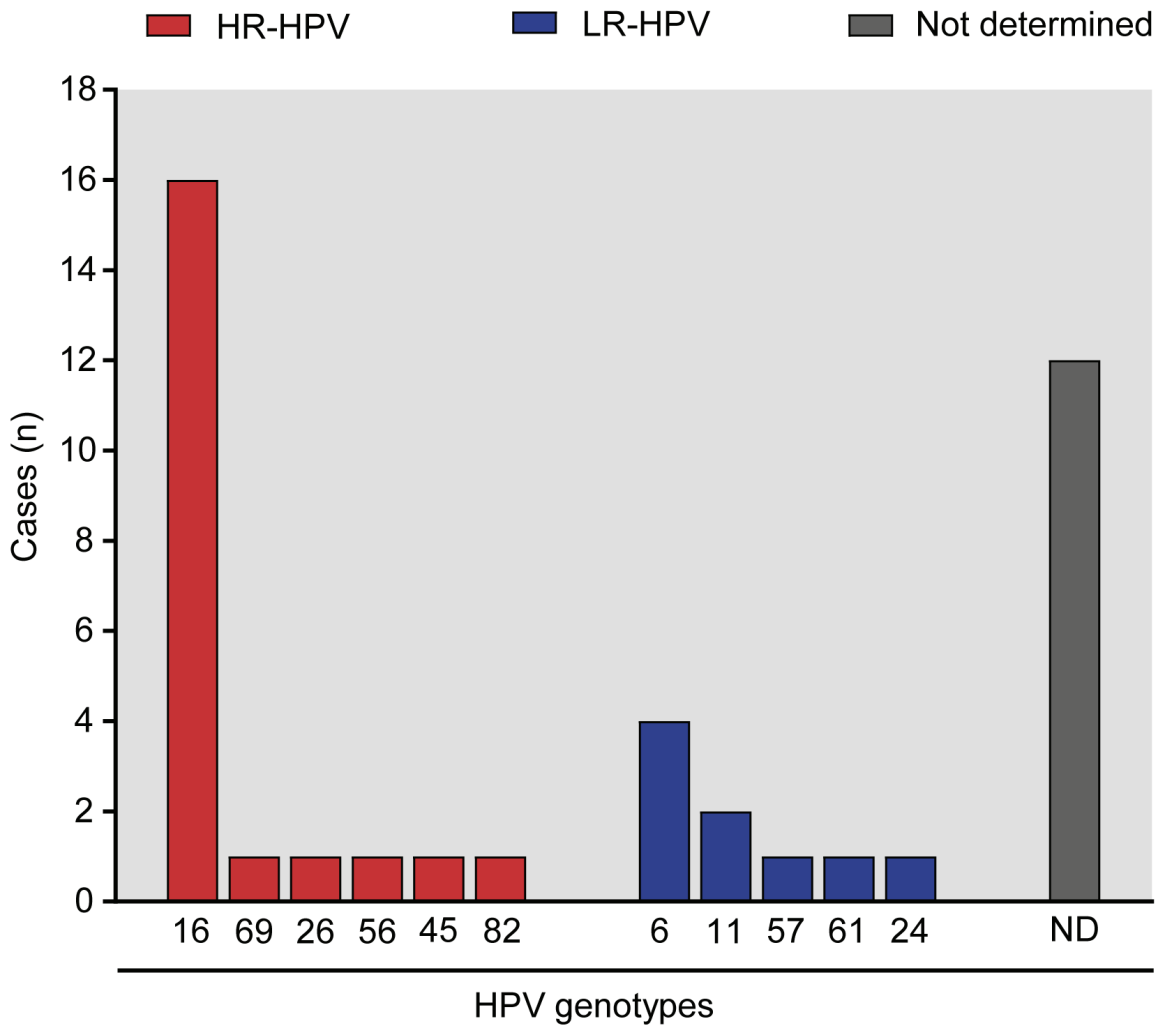
HPV genotypes identified in men with HPV urogenital infection. The bar graph illustrates the number of cases of identified genotypes in the patient cohort under study, classified as intermediate to high oncogenic risk (HR-HPV, depicted in red) and low oncogenic risk (LR-HPV, represented in blue) genotypes using the PCR-RFLP technique. The proportion of unclassified HPV genotype(s) using this method is indicated as ‘Not Determined’ (ND, shown in gray).

The median age of the 205 male participants enrolled was 35 years (95% CI: 34–36 years old). Thirty-nine out of the 205 individuals were HPV-positive revealing a significantly high prevalence of the infection (19.0%). We could successfully identify the HPV genotypes in 27 specimens, while in the remaining 12 samples this was not possible most likely due to low viral loads. Interestingly, single HPV genotype infections were predominantly identified (88.9%, 24/27), whereas multiple infections were detected in only 11.1% (3/27) of cases and consisted in infections of no more than two HPV genotypes. When analyzing the identified HPV genotypes, at least one HR-HPV genotype was detected in 74.0% (20/27) of the HPV+ patients, 17 of which were single infections and 3 multiple infections involving at least one HR- genotype (HPV-16/HPV-24; HPV-16/HPV-61 and HPV-56/HPV-82). LR-HPV were present as a single infection in 7 out of 27 cases (26.0%) and 2 LR-HPV cases were co-infecting with HPV-16 in multiple infections (HPV-16/HPV-24; HPV-16/HPV-61). **Figure 1** shows the number of genotypes cases detected individually either as single or multiple infections. It can be seen that HPV-16 was by far the most frequently detected genotype (n=16, 59.2%) followed by the LR-genotype HPV-6 (n=4, 14.8%).

### 3.2 Sperm quality in patients with high or low-risk HPV male urogenital infection

**Figure 2:**
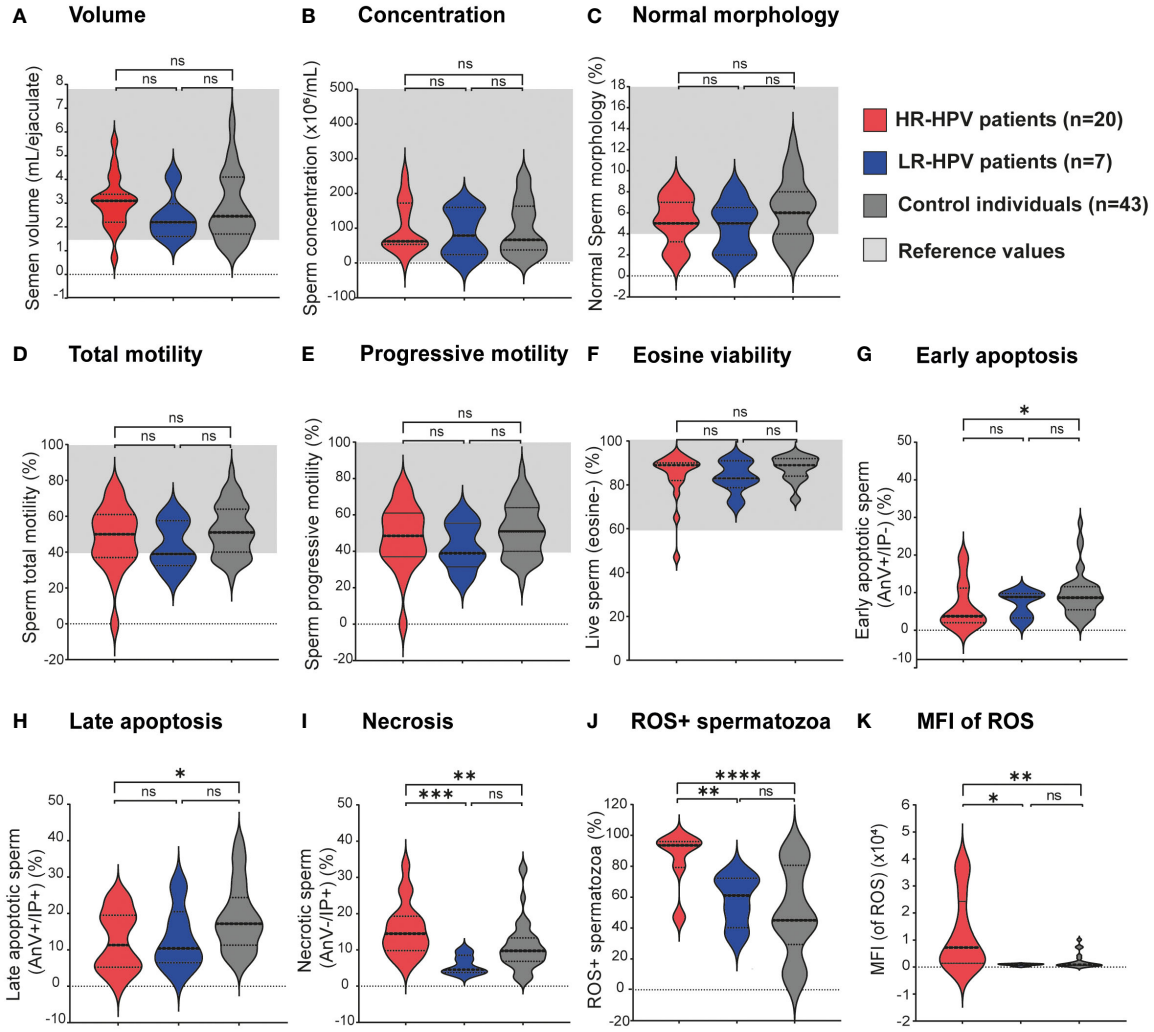
Sperm quality from patients bearing high or low-risk HPV male urogenital infections and non-infected control individuals. Violin plots display **(A–F)** classic sperm quality parameters analyzed following the WHO 2010 criteria, **(G–I)** the type of sperm death (apoptosis/necrosis) assessed by AnV/PI staining and flow cytometry, **(J)** frequencies of Reactive Oxygen Species (ROS)-positive spermatozoa and **(K)** ROS-dependent mean fluorescence intensity (MFI) values in spermatozoa evaluated by flow cytometry using the Dcfh-DA probe. Dotted lines indicate median and interquartile ranges and, for semen analysis parameters, reference ranges are shown in light gray shading. Comparisons were made between the following groups: control individuals (gray) without leukocytospermia and negative for all analyzed uropathogens; HR-HPV patients (red): all HPV-positive patients bearing infections by intermediate or high oncogenic risk genotypes; LR-HPV patients (blue): all HPV-positive patients bearing infections by low oncogenic risk genotypes. Comparisons were performed between each group and the control group, and *p*-values were calculated using the Mann-Whitney test (*p<0.05, **p<0.01 and ***p<0.001, ****p<0.0001). ns, not significant.

To investigate the potential impact of HR- and LR-HPV infections on male fertility, we analyzed sperm quality and sperm apoptosis/necrosis in HR- or LR-infected patients and control individuals. Patients with HPV urogenital infection were classified into two groups: one encompassing those individuals infected with high or intermediate risk genotypes (HR-HPV group) and another including patients infected with LR-HPV genotypes (LR-HPV group). Individuals from the same study population without any detected infection were used as controls. When assessing routine sperm parameters, including ejaculate volume, sperm concentration, total and progressive sperm motility, sperm morphology, and sperm viability (assessed by eosin staining), comparable values were observed between the control group and those infected with either HR-HPV or LR-HPV (**Figures 2A–F**). However, interesting differences were observed among groups under study when analyzing sperm apoptosis/necrosis (**Figures 2G–I**). While no differences were found in the frequencies of live spermatozoa between controls and HPV+ individuals (**Figure 2F**), significantly lower levels of early (AnV+PI-) and late (AnV+PI+) apoptotic spermatozoa were observed in men infected with HR-HPV than in controls (**Figures 2G, H**). Furthermore, HR-HPV infected individuals showed significantly higher levels of sperm necrosis (AnV-PI+) with respect to either LR-HPV infected or control individuals (**Figure 2I**).

### 3.3 Sperm ROS production in men bearing HR-HPV or LR-HPV urogenital infection

We then investigated whether urogenital infections with HR- or LR-HPV were associated with increased sperm ROS production. As shown in **Figure 2J**, significantly higher frequencies of ROS+ spermatozoa were observed in HR-HPV infected individuals (84.63 ± 18.65) with respect to either LR-HPV infected (57.86± 17.07) or control individuals (50.01 ± 29.02). Moreover, markedly higher Mean Fluorescence Intensity (MFI) values for sperm ROS production were observed in HR-HPV infected patients than in either LR-HPV-infected patients or control individuals (**Figure 2K**). These results indicate that male urogenital infection with HR-HPV associates with higher levels of sperm ROS production, either in terms of frequency of ROS producing cells or in the amounts of ROS produced by each cell. Interestingly, higher percentages of ROS+ dead spermatozoa (Dcfh-DA+IP+) were found in HR-HPV infected patients than in LR-HPV group, in which the highest proportion of ROS-producing sperm remained viable (Dcfh-DA+IP-) (data not shown). These results are consistent with the elevated levels of necrotic spermatozoa observed in samples from patients carrying HR-HPV.

### 3.4 Level of leukocyte subpopulations and cytokines in semen from patients with HR-HPV or LR-HPV urogenital infection

No differences in the counts of peroxidase-positive cells in semen between patients infected with either HR-HPV or LR-HPV and controls individuals were found (**Table 1**). Noteworthy, the assessment of peroxidase-positive cells detects neutrophils and activated macrophages neglecting other leukocyte subsets such as lymphocytes. To overcome this limitation, we additionally evaluated the levels of CD45+ cells in semen by flow cytometry, which is a panleukocytic biomarker that identifies all leukocyte subsets. In striking contrast to expectations, HR-HPV infected men displayed significantly lower semen CD45+ cell counts compared to control individuals; however, no such distinction was found for LR-HPV infected men (**Table 1**). Nevertheless, when assessing in further detail different leukocyte subpopulations, no significant differences in the levels of any particular cell subset (neutrophils, lymphocytes, or monocytes) were observed between patients infected with either HR- or LR-HPV and controls (**Table 1**). These results indicates that the decrease in the overall count of leukocytes was not due to a decrease in a specific subpopulation type, such as neutrophils, lymphocytes, or monocytes. Instead, the observed decrease was likely due to a general reduction in the number of all subsets of CD45+ cells.

**Table 1:** Total leukocytes and different leukocyte cell subsets in semen from patients bearing high or low-risk HPV male urogenital infections.

Total leukocytes and different leukocyte cell subsets	Control individuals (n=43)	HR-HPV patients (n=20)	p- value^a^	LR-HPV patient (n=7)	p- value^b^	p- value^c^
Peroxidase-positive cells (x10^5^/ml)	2.12 ± 2.84	1.01 ± 1.94	0.208	3.88 ± 6.2	0.929	0.489
CD45-positive cells (x10^5^/ml)	6.75 ± 10.3	1.83 ± 2.95	**0.019**	11.01 ± 15.2	0.707	0.205
PMN leukocytes (x10^3^/ml)	148.76 ± 214.6	121.07 ± 193.85	0.677	96.57 ± 182.12	0.356	0.282
Monocytes (x10^3^/ml)	64.14 ± 114.41	37.15 ± 63.86	0.671	61.44 ± 138.46	0.207	0.207
Lymphocytes (x10^3^/ml)	47.92 ± 98.86	10.96 ± 13.84	0.405	34.13 ± 40.78	0.615	0.183
T cells (x10^3^/ml)	16.88 ± 46.66	3.49 ± 5,33	0.349	25.7 ± 36.38	0.526	0.340
CD4 T cells (x10³/ml)	3,55 ± 9.78	1,97 ± 4.25	0.913	1.16 ± 1.75	0.803	0.538
CD8 T cells (x10^3^/ml)	2,98 ± 4.88	1.15 ± 2,33	0.084	1.02 ± 1,67	0.487	0.682
CD4/CD8 T cells ratio	1,44 ± 1.91	2.68 ± 2.67	0.160	1.6 ± 1,98	0.620	0.449
B cells (x10^3^/ml)	2.19 ± 4.64	3.23 ± 3.92	0.339	0.27 ± 0.28	0.531	0.268

HR-HPV patients: all HPV-positive patients presenting intermediate or high oncogenic risk genotypes; LR-HPV patients: all HPV-positive patients presenting low oncogenic risk genotypes; Control individuals: Individuals without leukocytospermia and negative for all analyzed uropathogens. Leukocyte population analyses were conducted using flow cytometry within gates defined on the total leukocyte cell population (CD45+). The subpopulations identified included polymorphonuclear leukocytes (PMN, CD11c-CD11b+), monocytes (CD14+CD11b-), and lymphocytes (CD11c-, CD11b-). T cells were characterized as CD3+ lymphocytes, with further subtyping into CD4 T cells (CD3+ CD4+ CD8-) and CD8 T cells (CD3+CD4-CD8+), along with B cells (CD3-CD20+). The results were reported as absolute leukocyte counts. p-values were calculated using the Mann-Whitney test. Comparisons were performed between HR-HPV group (^a^) or LR-HPV (^b^) and the control group. (^c^) Indicates comparisons between HR-HPV and LR-HPV groups. Data are shown as Mean ± SD. Bold numbers indicate statistically significant differences when p <0.05.

Intriguingly, when evaluating cytokine levels in seminal plasma, distinct profiles emerged based on the presence of urogenital infections caused by HR- or LR-HPV genotypes. In HR-HPV infected individuals, no significantly elevated levels of any cytokine analyzed (IL-8, IL-6, IL-1β, TNF, IFN-γ, IL-10, and IL-17A) was observed with respect to controls (**Table 2**). However, HR-HPV-infected patients showed significantly reduced semen levels of IL-6 and IL-1β than controls (**Table 2**). Conversely, LR-HPV-infected patients showed significantly increased levels of IL-1β than controls. Moreover, patients with LR-HPV infections showed significantly higher semen levels of IL-10 with respect to HR-HPV infected men, while the latter showed higher levels of semen IL-17 than LR-HPV infected patients (**Table 2**).

**Table 2:** Cytokine levels in semen from patients bearing hig or low-risk HPV male urogenital infections.

Cytokine (pg/mL)	Control individuals (n=43)	HR-HPV patients (n=20)	p- value^a^	LR-HPV patient (n=7)	p- value^b^	p- value^C^
IL-6	123.4 ± 135.6	52.43 ± 88.03	**0.028**	73 ± 69.2	0.659	0.201
IL-1β	7.51 ± 5.61	3.61 ± 3.83	**0.001**	20.11 ± 13.67	**0.007**	**<0.0001**
TNF	11.64 ± 10.75	9.83 ± 6.06	0.766	11.57 ± 6.62	0.419	0.441
IL-8	3379 ± 2193	2907 ± 1706	0.531	2023 ± 1269	0.163	0.283
INFγ	25.09 ± 18.66	24.94 ± 11.73	0.892	30.03 ± 7.19	0.465	0.302
IL-17	12.74 ± 10.81	16.19 ± 8.75	0.104	6.2 ± 6.03	0.213	**0.044**
IL-10	8.78 ± 6.1	7.76 ± 8.06	0.153	20.47 ± 18.82	0.058	**0.029**

HR-HPV patients: all HPV-positive patients presenting intermediate or high oncogenic risk genotypes; LR-HPV patients: all HPV-positive patients presenting low oncogenic risk genotypes; Control individuals: Individuals without leukocytospermia and negative for all analyzed uropathogens; p-values were calculated using the Mann-Whitney test. Comparisons were performed between HR-HPV group (^a^) or LR-HPV (^b^) and the control group. (^c^) indicate comparisons between HR-HPV and LR-HPV groups. Data are shown as Mean ± SD. Bold numbers indicate statistically significant differences when p<0.05.

### 3.5 Impact of HR-HPV infection, alone or in combination with other uropathogens, on semen quality and inflammatory markers

Subsequently, to study the effects of the presence of other urogenital infections in combination with HR-HPV on the different parameters analyzed, we categorized patients into those positive for HR-HPV and negative for all other infections analyzed (HR-HPV+ Coinf-) and those positive for HR-HPV as well as for at least one of the other uropathogens screened (HR-HPV+ Coinf+). When conventional sperm quality parameters were analyzed, no differences were observed between HR-HPV+ Coinf- or HR-HPV+ Coinf+ individuals and controls (**Supplementary Table 1**). Interestingly, higher frequencies of necrotic spermatozoa were detected in both HPV+ Coinf- and HR-HPV+ Coinf+ patients with respect to controls. However, this increase was statistically significant only in HR-HPV+Coinf+ individuals (*p*=0.033) whereas showing a tendency in HR-HPV+ Coinf- individuals (*p*=0.071). Besides, significantly decreased semen levels of IL-6 and IL-1β were detected in HR-HPV patients regardless of the presence of coinfections, while significantly reduced levels of IL-10 were only detected in HR-HPV+Coinf- patients (**Supplementary Table 1**). Interestingly, significantly higher frequencies of ROS+ spermatozoa were observed in HR-HPV infected patients either with or without coinfections than control individuals. Moreover, higher frequencies of ROS+ dead spermatozoa (Dcfh-DA+IP+) were detected in both HR-HPV+Coinf- and HR-HPV+Coinf+ patients with respect to controls (**Supplementary Table 1**). When assessing leukocytes in semen, a significant reduction in CD45+ cell levels was observed in HR-HPV+ Coinf+ patients with respect to controls, while no changes in the leukocyte subsets were observed (**Supplementary Table 1**). These results show that the infection with HPV-HR itself associates with increased sperm ROS production and necrosis in the absence of semen inflammation, whereas the co-infection with other common uropathogens may slightly worsen some of these effects.

Taken together, our data indicate that HPV male urogenital infection neither associates with prominent semen inflammation nor major detrimental effects on sperm quality. On the contrary, significantly reduced leukocyte recruitment and decreased levels of inflammatory cytokines were observed, especially when the infection was caused by HR-HPV genotypes.

## 4 Discussion

The study of sexually transmitted infections in men and their consequences on sperm quality and male fertility potential has gained increasing attention during the last decades (Rivero et al., 2023). Although HPV is the most prevalent viral uropathogen worldwide, HPV infection of the male urogenital tract particularly constitutes an emerging field of study encompassing several unanswered queries (Zou et al., 2022). In the present work, we analyzed urogenital infection with HPV in a cohort of sexually active adult men attending a urology clinic, with special focus on the effects of the infection caused by high-risk or low risk HPV genotypes on semen inflammation and sperm quality. A significant prevalence (19.0%) of urogenital HPV infection was observed and, notably, infections by HR-HPV genotypes were by far more prevalent than those by LR-HPV. Noteworthy, high-risk HPV-16 was the genotype most frequently found in the semen of these patients, followed by HPV-6. These findings align with a recent meta-analysis that also identified HPV-16 as the most prevalent genotype in men, followed by HPV-6, similar to women (Bruni et al., 2023). HPV-16 is commonly associated with various HPV-related cancers, while HPV-6 is a prevalent cause of genital warts, and both are preventable through vaccination. It is important to note that our study conducted HPV detection at a single time point and HPV is not continuously shed in semen, leaving open the possibility, HPV DNA may not be present in all ejaculates of an HPV-infected patient (Fedder et al., 2019). Since there are no official statistics on HPV male infection in Argentina, our data represent valuable evidence for the design of public health policies and the improvement of clinical care of patients.

Few studies have compared the impact of male urogenital infections by HR- or LR-HPV genotypes on sperm quality, and they have reported dissimilar results (Luttmer et al., 2016; Damke et al., 2017; Boeri et al., 2019; Moreno-Sepulveda and Rajmil, 2021). Two studies indicated that HR-HPV+ patients show significant alterations in sperm quality such as impaired semen viscosity, sperm progressive motility, and increased sperm DNA fragmentation, when compared with those infected by LR-HPV (Damke et al., 2017; Boeri et al., 2019). However, contradicting findings were reported by other researchers (Luttmer et al., 2016). Moreover, Canarella et al. reported no significant differences in sperm quality between control individuals and LR-HPV infected men (Cannarella et al., 2022). Supporting these findings, our results show that male urogenital infection by either HR-HPV or LR-HPV does not impair most sperm quality parameters routinely included in WHO, 2010 guidelines for semen analysis. However, using a more sensitive assay our findings revealed distinct patterns of sperm cell death. Specifically, we observed elevated levels of necrotic spermatozoa in individuals with HR-HPV infection. The observed increase in sperm necrosis raises the question whether overall nuclear integrity is actually preserved in spermatozoa from individuals infected with HR-HPV. While not all reports have identified sperm DNA fragmentation in HPV-positive individuals (Kaspersen et al., 2013; Cortés-Gutiérrez et al., 2017), we may speculate that the cause of the elevated sperm necrosis found in the HR-HPV group could be linked to higher sperm DNA fragmentation previously reported by some authors (Connelly et al., 2001; Boeri et al., 2019; Capra et al., 2022). Among DNA abnormalities in spermatozoa, fragmentation is the most common, particularly in infertile subjects (Agarwal et al., 2022). Currently, compelling evidence indicates that spermatozoa with fragmented DNA can be viable, motile, morphologically normal, and capable of fertilizing an oocyte (Tamburrino et al., 2012). Moreover, the oocyte can repair sperm DNA damage after fertilization; however, it depends on the degree of DNA damage in the spermatozoa as well as on the quality of the oocyte. The combination of these factors will determine the fate of embryo development, pregnancy outcome, and health of the offspring conceived either naturally or through Assisted Reproductive Technology (ART) (Tamburrino et al., 2012). In that regard, our findings showing increased sperm necrosis in men infected with HR-HPV could be related to the lower rates of clinical pregnancy observed in women that underwent intrauterine insemination with sperm from HPV+ men (Garolla et al., 2016; Busnelli et al., 2023; Sucato et al., 2023; Tramontano et al., 2023).

Another interesting finding from our study is the observed increase in sperm ROS production in HR-HPV+ patients. Moreover, higher frequencies of ROS+ dead spermatozoa were shown by HR-HPV infected individuals with respect to those infected with LR-HPV genotypes. It is well known that oxidative stress is highly detrimental to spermatozoa and one of the main causes of sperm necrosis (Aitken et al., 2022; Sharma et al., 2022; Hussain et al., 2023). Thus, it is possible that HR-HPV infection predisposes to higher rates of sperm death due to increased oxidative stress, partially explaining the higher percentage of spermatic necrosis found in HR-HPV infected patients. Within semen, leukocytes are the predominant source of ROS, followed by spermatozoa, particularly those that are immature, immotile, and/or dysfunctional, or under the influence of elevated levels of inflammatory cytokines (Sharma et al., 2022; Paira et al., 2023). Leukocytospermia has been suggested as an indicator of inflammation or potential infection in the urogenital tract. It is defined as an elevation in peroxidase-positive cells in semen above 1x10^6^ leukocytes/ml of semen. Nevertheless, its direct association with confirmed genital tract infections is controversial (Bachir and Jarvi, 2014; Sharma et al., 2022). As our data showed no significant increases in the levels inflammatory cytokines assayed and even decreased counts of CD45+ cells (leukocytes) in semen, with no differences in any particular cell subset, the heightened levels of sperm ROS observed in these patients could be attributed to dysregulated sperm ROS production stemming from increased metabolite generation and/or a decline in their antioxidant capacity (Aitken et al., 2022). Indeed, certain *in-vitro* studies have associated the presence of HR-HPV with oxidative stress and subsequent cellular DNA damage (Williams et al., 2014; Cruz-Gregorio et al., 2021). Moreover, Pérez-Soto et al. showed reduced levels of antioxidant enzymes and high levels of sperm lipoperoxidation in semen from HR-HPV infected men than in uninfected individuals (Pérez-Soto et al., 2022).

Interestingly, it has been shown that HR-HPV, especially HPV-16, can inhibit innate immune responses, thereby facilitating viral persistence (Zhou et al., 2019). Indeed, it has been shown that the E6/E7 oncoproteins of HR-HPV can block several signaling pathways involved in the activation of innate immunity cells and the secretion of cytokines and antigen presentation (Amador-Molina et al., 2013; Bordignon et al., 2017). In our study, we observed a distinct cytokine profile in semen depending on whether the infection was caused by HR- or LR-HPV genotypes. Upon assessing cytokine levels in semen, we found reduced levels of the inflammatory cytokine IL-6 in seminal plasma from HR-HPV patients compared with non-infected controls. These findings are in line with those reported by Bonin-Jacob et al., who reported reduced secretion of IL-6 by cervical cells from HR-HPV+ women with respect to HPV negative women (Bonin-Jacob et al., 2021).

Remarkably, when segregating HPV+ patients into HR- or LR-HPV infected individuals, significant decreased semen levels of IL-1β were observed in HR-HPV infected patients with respect to control individuals. Conversely, LR-HPV infected patients exhibited significantly increased semen levels of IL-1β than control individuals. IL-1β is a potent proinflammatory cytokine produced as the first-line defense during viral infections. It plays a crucial role linking innate immunity with subsequent adaptive immune response by inducing leukocyte migration and T cell polarization (Orzalli et al., 2018). Due to its pleiotropic physiological effects, IL-1β production is finely regulated at transcriptional, post-translational, and secretion levels. The modulatory effect of HR-HPV infection on IL-1β expression has already been described in *in vitro* experimental models (Niebler et al., 2013; Ainouze et al., 2018). Ainouze et al. demonstrated that the expression of early genes of HPV-16 in human keratinocytes led to the inhibition of IL-1β gene transcription. They further showed that the expression of the oncoprotein E6 of HPV-16 inhibited IRF6 transcription, thus blocking IL-1β production (Ainouze et al., 2018). Additionally, Niebler et al. proposed a novel post-translational regulatory mechanism for pro-IL-1β, where HPV-16 E6 facilitates the degradation of IL-1β via the ubiquitin ligase E6-AP and the p53-dependent proteasomal pathway (Niebler et al., 2013). This dysregulation of IL-1β expression was also found in HPV+ tissues at different stages of carcinogenesis and in persistent genital warts induced by the virus (Niebler et al., 2013; Castagnino et al., 2022). Consequently, to the best of our knowledge, our study provides the first evidence that HR-HPV may also induce dysregulation of IL-1β in semen of men bearing HR-HPV urogenital infection. Our data highlight that this effect is exclusively associated with the infection by HR-HPV genotypes, suggesting that cells infected by LR-HPV could maintain their capacity to generate IL-1β. This regulation is not only critical for HPV to successfully evade host immunity, thus promoting oncoviral persistence, but also for other infections since it indirectly increases host susceptibility. Remarkably, the observed increased semen levels of IL-1β were accompanied by increased levels of the anti-inflammatory cytokine IL-10 in LR-HPV infected patients, but not in those infected by HR-HPV, in whom a local immunosuppressive profile was also observed revealed by decreased semen levels of IL-6, IL-1β and leukocytes. Our findings are in agreement with previously reported data showing that IL-10 secretion by cervical cells increases after HPV infection (Berti et al., 2017). Interestingly, our data also revealed decreased semen levels of IL-17A in LR-HPV+ individuals with respect to HR-HPV+ individuals. Some authors have proposed that Th17 cells and IL-17 may have a negative role during HR-HPV cervical infection, contributing to the severity of associated lesions (Xue et al., 2018). Up to our knowledge, no prior data about semen levels of IL-10 and IL-17A in LR-HPV or HR-HPV male urogenital infection have been reported.

While a limitation of our study is the small number of samples analyzed, particularly in the LR-HPV group, its strength lies in the comprehensive analysis of several parameters beyond the classic and routinely performed semen analysis. Our experimental approach offers a more thorough understanding of how HR- and LR-HPV infections may affect male reproductive health. Our findings raise questions about the importance of identifying the HPV genotype in men and whether distinguishing between infections by LR-HPV and HR-HPV has clinical relevance. Current clinical protocols may struggle to differentiate HPV-positive males from uninfected individuals given that conventional semen analysis often overlooks substantial changes in sperm quality parameters. However, a more comprehensive assessment revealed increased sperm necrosis and a local immunosuppressive environment, potentially facilitating persistent HPV infection and co-infection with other uropathogens, thus promoting transmission to sexual partners. Moreover, categorizing HPV solely based on its oncogenic potential may be limiting since HR-HPV not only disrupts the cell cycle and induces cellular transformation but also affects cellular metabolism and modulates immune responses. Therefore, a more thorough evaluation, including genotyping, is essential for males seeking care at urology and fertility clinics, since the specific HPV genotype may influence the course of the infection and its consequences on reproductive health.

## Data availability statement

The raw data supporting the conclusions of this article will be made available by the authors, without undue reservation.

## Ethics statement

The studies involving humans were approved by Institutional Ethics Committee from Centro Médico Oulton-Romagosa, Córdoba, Argentina. The studies were conducted in accordance with the local legislation and institutional requirements. The participants provided their written informed consent to participate in this study.

## Author contributions

CO: Conceptualization, Writing – original draft, Writing – review & editing, Formal analysis, Investigation, Methodology. DP: Methodology, Writing – review & editing. AO: Writing – review & editing, Investigation. JO: Writing – review & editing, Investigation. ADT: Writing – review & editing, Formal analysis, Investigation, Methodology. RIM: Formal analysis, Investigation, Methodology, Writing – review & editing. RDM: Writing – review & editing, Funding acquisition, Investigation. CC: Writing – review & editing, Conceptualization, Investigation. VR: Conceptualization, Investigation, Writing – review & editing, Funding acquisition, Project administration, Supervision, Writing – original draft.
