# Supplementary figures and images for: Impact of high-risk and low-risk human papillomavirus infections on the male genital tract: effects on semen inflammation and sperm quality
Source: Front Cell Infect Microbiol. 2024 Aug 23;14:1420307. doi: 10.3389/fcimb.2024.1420307 (PMC11385601; doi:10.3389/fcimb.2024.1420307)

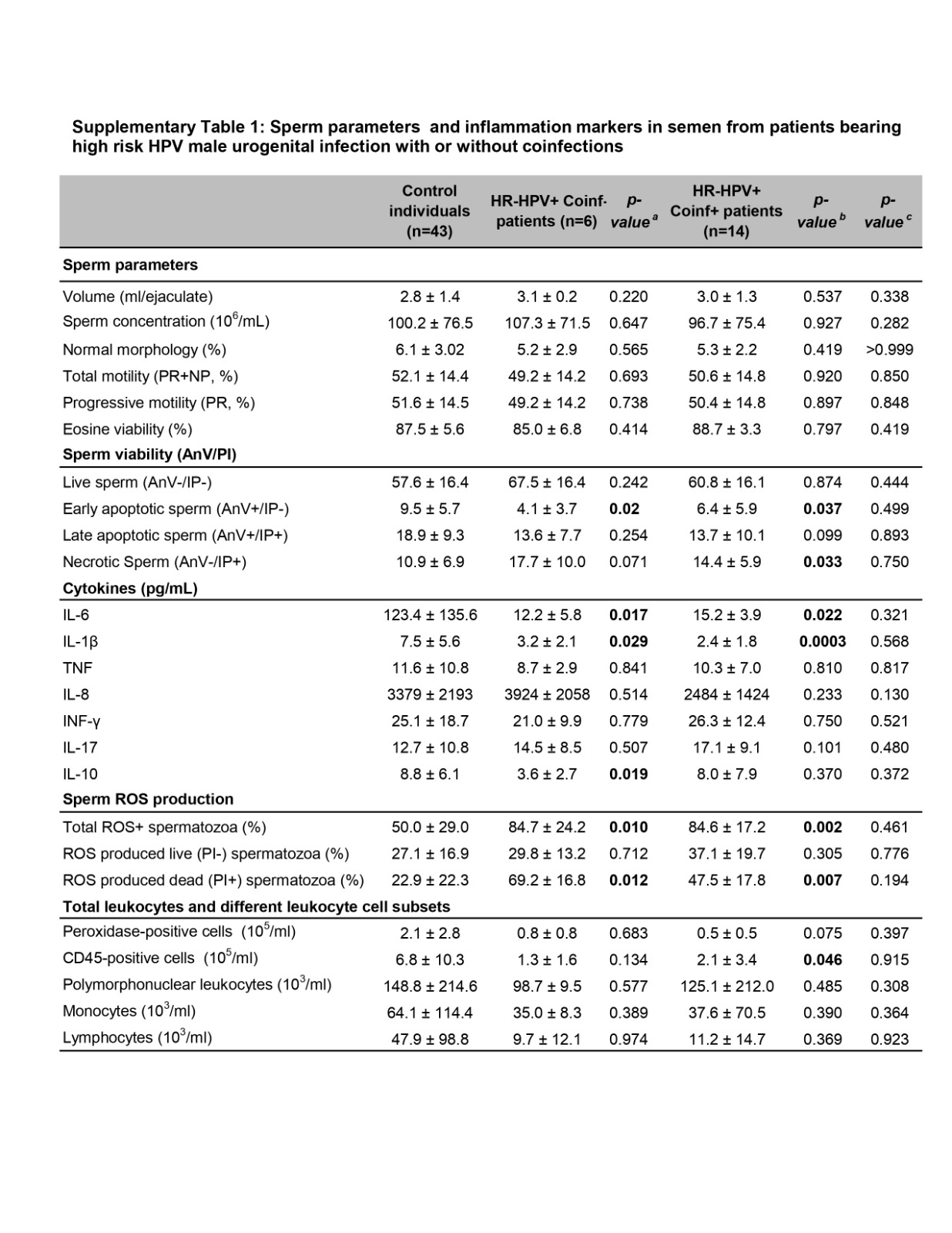

Supplement: Supplementary file 1 [file Image_1.jpeg]
